# Alzheimer's Disease: APP, Gamma Secretase, APOE, CLU, CR1, PICALM, ABCA7, BIN1, CD2AP, CD33, EPHA1, and MS4A2, and Their Relationships with Herpes Simplex, *C. Pneumoniae*, Other Suspect Pathogens, and the Immune System

**DOI:** 10.4061/2011/501862

**Published:** 2011-12-29

**Authors:** Chris Carter

**Affiliations:** PolygenicPathways, Flat 2, 40 Baldslow Road, Hastings, East Sussex TN34 2EY, UK

## Abstract

Alzheimer's disease susceptibility genes, APP and gamma-secretase, are involved in the herpes simplex life cycle, and that of other suspect pathogens (*C. pneumoniae*, *H. pylori*, *C. neoformans*, *B. burgdorferri*, *P. gingivalis*) or immune defence. Such pathogens promote beta-amyloid deposition and *tau* phosphorylation and may thus be causative agents, whose effects are conditioned by genes. The antimicrobial effects of beta-amyloid, the localisation of APP/gamma-secretase in immunocompetent dendritic cells, and gamma secretase cleavage of numerous pathogen receptors suggest that this network is concerned with pathogen disposal, effects which may be abrogated by the presence of beta-amyloid autoantibodies in the elderly. These autoantibodies, as well as those to nerve growth factor and *tau*, also observed in Alzheimer's disease, may well be antibodies to pathogens, due to homology between human autoantigens and pathogen proteins. NGF or *tau* antibodies promote beta-amyloid deposition, neurofibrillary tangles, or cholinergic neuronal loss, and, with other autoantibodies, such as anti-ATPase, are potential agents of destruction, whose formation is dictated by sequence homology between pathogen and human proteins, and thus by pathogen strain and human genes. Pathogen elimination in the ageing population and removal of culpable autoantibodies might reduce the incidence and offer hope for a cure in this affliction.

## 1. Introduction

Hundreds of genes have been implicated in Alzheimer's disease, many of which can be grouped into discrete signalling networks and pathways relevant to the various subpathologies, risk factors, and biochemistry of Alzheimer's disease. Many of the environmental risk factors associated with Alzheimer's disease, including infectious agents (herpes simplex, *chlamydia pneumonia*, and *Borrelia burgdorferi*) as well as Vitamin A deficiency, hypercholesterolaemia, hyperhomocysteinaemia or folate deficiency, oestrogen depletion, cerebral nerve growth factor (NGF) deprivation, diabetes, cerebral hypoperfusion (leading to hypoxia and hypoglycaemia) or are able to promote cerebral beta-amyloid deposition (in the absence of any particular gene variant) in animal models [1]. KEGG pathway and other analyses of the multiple genes implicated in Alzheimer's disease have shown that subsets of susceptibility genes can be grouped into networks that are relevant to each of these amyloidogenic pathways (e.g., bacterial and viral entry pathways [1, 2], cholesterol/lipoprotein function [3, 4], growth factor signalling [5], folate and homocysteine pathways [6], insulin signalling [7], and steroid or Vitamin A metabolism [8, 9]). A large number of genes are also related to the immune network [10] (see http://www.polygenicpathways.co.uk/alzkegg.htm and a recent review for further details [1]). These gene subsets are thus related to multiple external factors that are each able to promote beta-amyloid deposition, suggesting that certain genes are related to the causes of Alzheimer's disease, (agents able to provoke beta-amyloid deposition) rather than (and as well as) to the underlying pathology of the disease itself.

Several studies have implicated the herpes simplex virus in the aetiology of Alzheimer's disease [11–13]. Viral DNA is found in amyloid plaques [14], which are also heavily enriched in proteins used by the virus during its life cycle, as well as in proteins related to the immune network [15], and Immunoglobulin IgM, but not IgG seropositivity for herpes simplex is predictive of the subsequent development of Alzheimer's disease [16]. IgM seropositivity is indicative of viral reactivation which again can be induced by several of the risk factors relevant to Alzheimer's disease and its underlying genetic pathways (e.g., NGF deprivation, 17-beta oestradiol, hypoxia, or fever and interleukin 6 activation, with the latter being common and general consequences of infection [1]).

Along with herpes simplex, a number of other pathogens have been implicated in Alzheimer's disease and its associated pathologies. The viral, bacterial, spirochete, and fungal pathogens implicated in dementia or Alzheimer's disease are referenced at (http://www.polygenicpathways.co.uk/alzenvrisk.htm) and include HHV-6, *Chlamydia pneumoniae*, *Helicobacter pylori*, periodontal pathogens involved in gum disease [17], *Borrelia burgdorferi*, and *Cryptococcus neoformans*. HIV-1 is also able to provoke dementia with Alzheimer's disease pathology [18]. Of these, *H. pylori* eradication has been reported to improve performance and increase lifespan in Alzheimer's disease patients [19], while two case reports indicated virtually complete recovery from long-term (3 years) misdiagnosed dementia/Alzheimer's disease following antifungal treatment for *C. neoformans* infection [20, 21]. Many of these pathogens including herpes simplex, HHV-6, *C. Pneumoniae*, *H. pylori* and the periodontal pathogen, *P. Gingivalis*, have also been implicated in atherosclerosis [22–25], while *C. neoformans* infection in rabbits induces an increase in neutrophil superoxide production, plasma lipid peroxidation, and an increase in inflammatory cells, forerunners of atherosclerosis [26]. Atherosclerosis of the carotid arteries, or of the circle of Willis and leptomeningeal arteries, is a significant predictor of risk in dementia or Alzheimer's disease and correlates with Alzheimer's disease pathology  [27, 28]. Cerebral hypoperfusion (hypoglycaemia, hypoxia, ischaemia, or carotid occlusion) or other factors linked to atherosclerosis (e.g., high cholesterol or homocysteine levels) are also able, *per se*, to induce cerebral beta-amyloid deposition in animal models (see above).

Genomewide association studies (GWAS) have now identified a subset of genes which, along with **APOE4 **[29], contribute a high proportion of genetic risk. These include clusterin (**CLU**), phosphatidylinositol-binding clathrin assembly protein (**PICALM**) and complement receptor 1 (**CR1**) as well as the ATP cassette transporter **ABCA7**, Bridging integrator **BIN1**, a CD2-associated protein (**CD2AP**), **CD33**, ephrin A1 (**EPHA1**), and a membrane-spanning 4-domains, subfamily A (MS4A) cluster recently honed down to **MS4A2**, although other genes within this cluster may also be relevant [30, 31].

As discussed below, the major Alzheimer's disease genes implicated by the recent GWAS data, as well as **APP** and **gamma secretase**, and previous GWAS results are majoritarily involved in pathogen entry and defence, particularly in relation to herpes simplex, but also to other relevant pathogens, and in the immune network. This suggests that genes, pathogens, and the immune system act together to cause Alzheimer's disease, and that a focus on pathogen detection and elimination should be a priority in the ageing at risk population.

## 2. Methods

**Figure 1:**
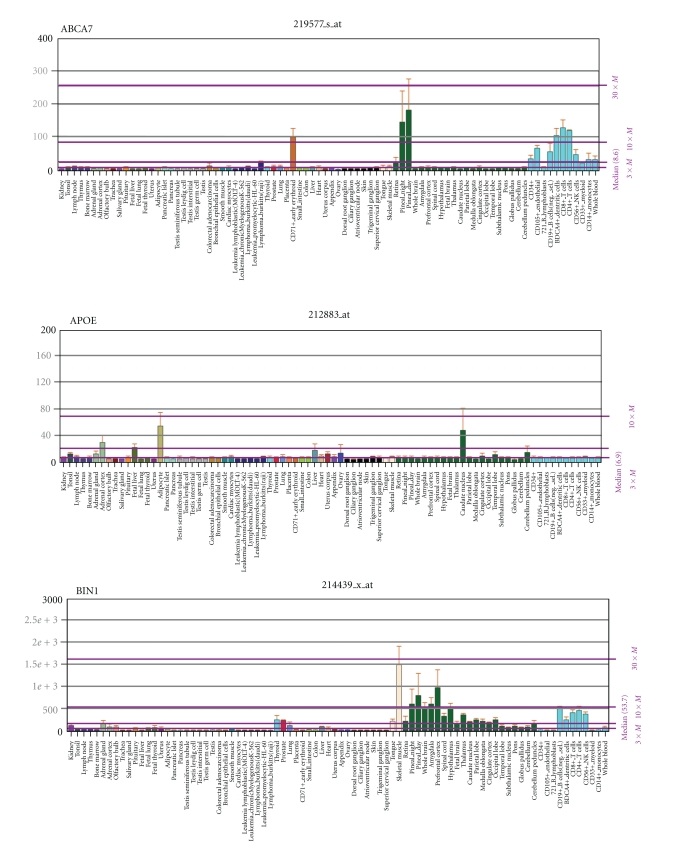

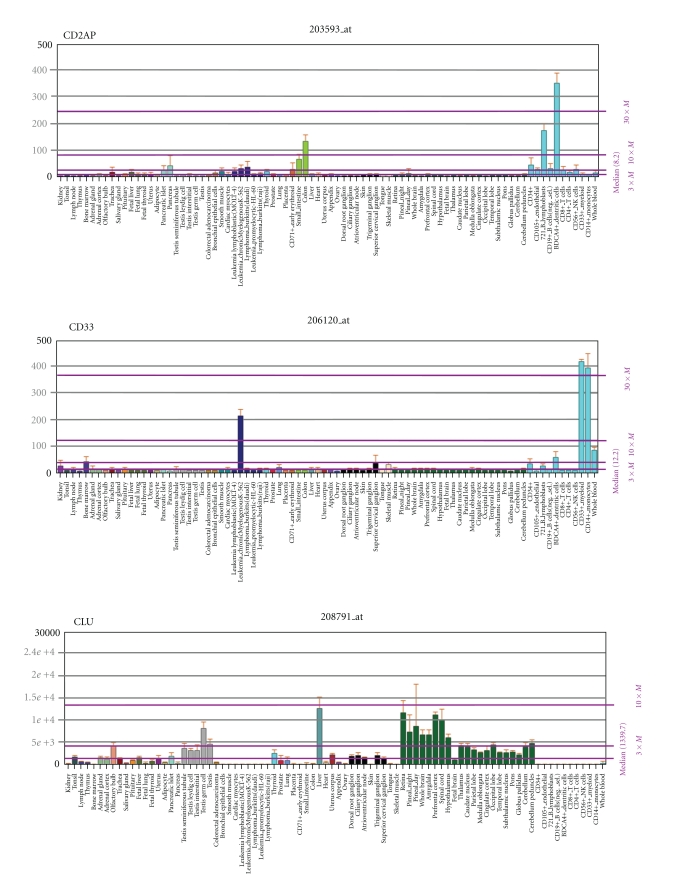

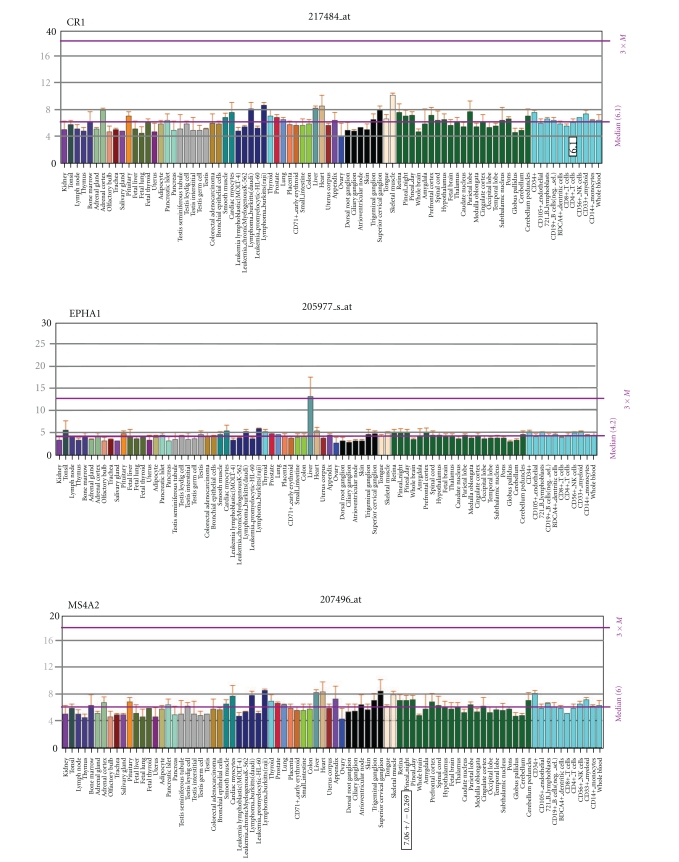

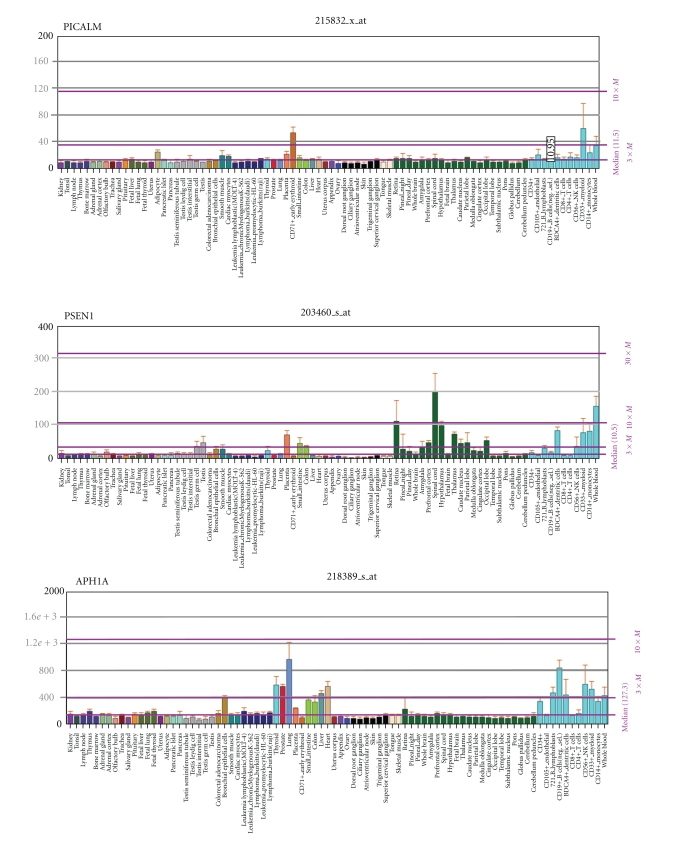

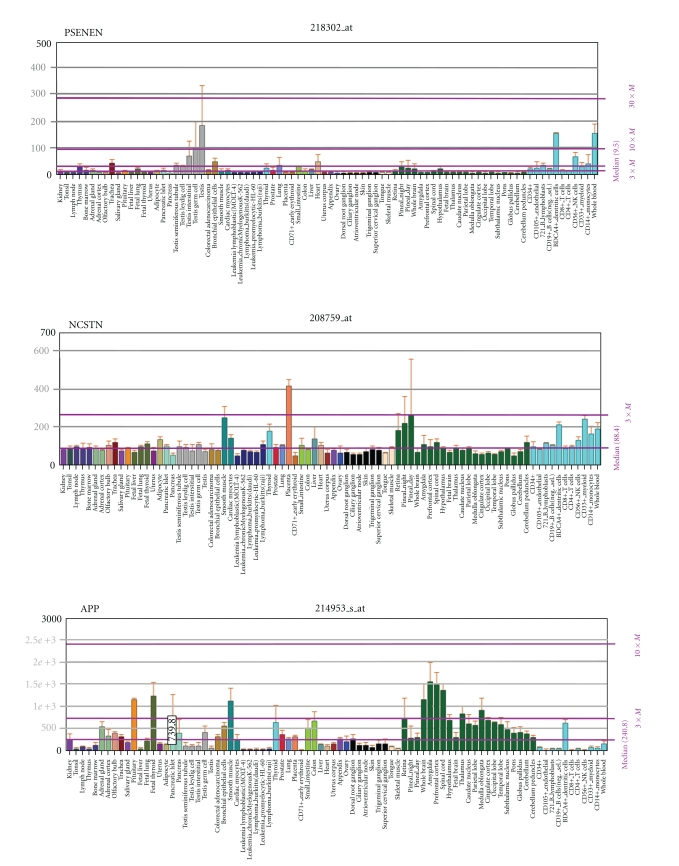
The mRNA distribution of the major genes derived from GWAS in Alzheimer's disease, as well as that of APP and gamma-secretase components. Data are from the BioGps website.

The genes identified in a number of recent genomewide association studies are available at the GWAS repository at the National Human Genome Research Institute http://www.genome.gov/gwastudies/ [32] and, along with pre-GWAS genes and environmental risk factors, at http://www.polygenicpathways.co.uk/alzenvrisk.htm. The genes returned from very large sample sets (*N* > 10,000) include **ABCA7**, **APOE**, **BIN1**, **CD2AP, CD33, CLU, CR1, EPHA1, MS4A2, MS4A4A, MS4A4E, MS4A6A**, and **PICALM** whose properties in relation to diverse pathogens were identified by literature survey. While it is recognised that such genes, particularly **APOE**, **ABCA7**, **CR1**, and **clusterin**, which are involved in lipoprotein function and/or amyloid processing (see below), may exert effects on other relevant branches of Alzheimer's disease pathophysiology, the focus of this paper is on pathogens and the immune system, which appear to be the common factors integrating this network. Throughout the text, these and other genes implicated in Alzheimer's disease from the GWAS and pre-GWAS era are highlighted in bold and appended to the various processes in which they are involved (derived from a KEGG pathway analysis of these genes http://www.polygenicpathways.co.uk/alzkegg.htm) Herpes simplex binding proteins, and key interactors, currently numbering over 450, are stocked and referenced at http://www.polygenicpathways.co.uk/herpeshost.html. KEGG pathway analysis of this interactome is provided at http://www.polygenicpathways.co.uk//HERPESKEGG.htm. Expression data are provided in Figure 1 and are also hyperlinked to the BioGPS webserver http://www.biogps.gnf.org/, which provides general gene information and mRNA expression profiles for most human genes, based on custom arrays from 79 human issues [33, 34]. Predicted B-cell epitopes from human beta-amyloid (1–42), nerve growth factor (NP_002497.2), or the microtubule protein, ***tau ***(NP_001116538.2) were identified using the BepiPred server http://www.cbs.dtu.dk/services/BepiPred/ [35] and their sequences compared with pathogen proteomes (*Borrelia burgdorferi*, *C. neoformans*, *Helicobacter pylori*, herpes viruses HSV-1, HSV-2, HHV-6, and the cytomegalovirus (HHV-5)) using the NCBI BLAST server (Protein versus protein: BlastP) [36].

## 3. Results

### 3.1. The Complement System (ABCA7, CR1, CLU, CD2AP, and Beta-Amyloid) Figure 2

**Complement receptor 1** (highly expressed in myeloid **CD33**+ cells (bone marrow) http://www.biogps.org/#goto=genereport&id=1378/) is a receptor for herpes simplex, adenovirus 5, the influenza virus and HIV-1, as well as for a number of other pathogens, including *P. gingivalis*, *C. neoformans*, *Streptococcus pneumoniae, Staphylococcus aureus*, and the malaria parasite, *Plasmodium falciparum* [37–43] and is a general clearance receptor for complement opsonised pathogens [44]. **Clusterin**, predominantly expressed in brain, liver, and testis, (http://www.biogps.org/#goto=genereport&id=1191/) is a ligand for the lipoprotein receptor, megalin (**LRP2)** that is involved in beta-amyloid clearance, and also a complement inhibitor that prevents the formation of the membrane attack complex, a channel that is inserted into pathogen membranes, killing them by lysis [45]. This complex is also seen in Alzheimer's disease neurones [46, 47]. The herpes simplex virus interacts with other members of the complement cascade, by binding to the complement component and **CR1** ligand, C3 and its derivatives and to CD59, a further inhibitor of the formation of the complement membrane attack complex (see review) [48]. *C. pneumoniae* interacts with this pathway by binding to properdin (CFP), a protein that stabilises the complement C3 and C5 convertase and contributes to the formation of the membrane attack complex [49]. CD59 is also incorporated into chlamydial inclusion bodies [50]. Complement component C3 binds to melanins derived from *C. neoformans* [51] and cryptococcal capsules bind to C3 and activate the alternative complement pathway [52]. Complement component C3 also binds to the bacterial surface of *H. pylori*, and the complement pathway is involved in bactericidal effects against this pathogen [53]. *P. gingivalis* also uses complement receptor 3 (an integrin complex of integrin, alpha M/integrin, beta 2 (ITGAM/ITGB2)) for entry [54], and herpes simplex glycoprotein C also binds to this complex [55] as does *C. neoformans* [56], while ITGB2 is involved in *C. pneumoniae* entry in human coronary artery endothelial cells [57]. This macrophage complement receptor, also known as MAC-1, generally mediates the phagocytosis of pathogens coated with complement C3 derivatives [58]. T. C3 also binds to *P. gingivalis* although the pathogen has devised an elegant escape strategy involving digestion of complement components C3, **C4**, and C5 by bacterial secreted proteases, known as gingipains [59].

The complement inhibitor CD59 is also a ligand for CD2, and CD59 activation of this receptor, presumably involving **CD2AP**, activates T cell receptor signalling resulting in the secretion of interleukins (**IL1A**, IL2 and **IL6**) and granulocyte macrophage colony stimulating factor (CSF2) [60, 61].

**ABCA7** plays a role in the complement-mediated activation of phagocytosis in macrophages. Complement component C1q, which binds to IgM or IgG complexed antigens (relevant to most pathogens), binds to macrophage calreticulin and **LRP1** and C1q binding to macrophages markedly increased the expression of both **LRP1** and **ABCA7**, effects which enhance the phagocytic abilities of macrophages [62]. C1q also binds to complement receptor **CR1**, an effect involved in the immune clearance of opsonised pathogens [63]. C1q also binds to beta-amyloid and is involved in amyloid-related complement activation [64].

### 3.2. Clathrin-Mediated Endocytosis (BIN1, CLU, CD2AP, PICALM) Figure 3

Mammalian surface receptors are endocytosed, via clathrin-dependent or independent processes (KEGG: **ADRB1, ADRB2, BIN1, CAV1, CD2AP, CLU, DNM2, HLA-A, HSPA1B, LDLR, NTRK1, PICALM**) and either recycled or tagged for destruction by the ubiquitin/proteasome system **(KEGG: UBD, UBE2I UBQLN1, UCHL1**) or by lysosomes (KEGG: **ABCA2, ARSA, ARSB, CTSD, CTSS, NPC1, NPC2, LIPA**). Early endosomes receive traffic from the cell surface, which is transferred to late endosomes for traffic to lysosomes. Late endosomes also receive traffic from the trans-Golgi network used to synthesise proteins and from phagocytic pathways (KEGG: **CTSS**, **DLD, DLST, DNM2, GAB2, HLA-A, HLA-DRB1, MPO, NOS1, OLR1, PIK3R1, PSK1, TAP2, TLR2, TLR4**). Endosomal traffic moves along the microtubule (**GSK3B, MAPT, TTLL7**) or actin/myosin (**MYH8, MYH13**) networks via dynein/dynactin (**DM2, DNMBP**) or kinesin (**KIF18B, KIF20B, KNS2**), related motors and Rho GTPases, and vacuolar sorting proteins (**SORCS1, SORCS2, SORCS3, SORL1**) *inter alia *[65]. These processes are usurped by many viruses and other pathogens to gain access to cells and to various intracellular compartments, while the lysosomal or proteasomal pathways may be used to destroy pathogen proteins [66].

Clathrin-mediated endocytosis is one of several processes used by *Helicobacter pylori*, herpes simplex, and many other viral, bacterial and fungal pathogens to gain entry to cells [67–69].

**PICALM**, expressed primarily in myeloid and dendritic cells of the immune network http://www.biogps.org/#goto=genereport&id=8301/, plays a key role in clathrin-related endocytosis, binding to clathrin heavy chains (CLTC and CLTCL1), and recruiting the clathrin and adaptor protein 2 (AP-2) to the plasma membrane. The AP-2 complex is a heterotetramer consisting of permutations of two large adaptins (alpha (AP2A1, AP2A2)) or beta (AP2B1), a medium adaptin (AP1M1, AP1M2), and a small adaptin (sigma AP2S1). **PICALM** controls the endocytosis of the cation-independent mannose-6-phosphate IGF2 receptor (IGF2R) [70], one used by Herpes simplex for entry and cell-to-cell transmission [71] and by *C. pneumoniae* for cellular entry [57]. IGF2R is also a component of late endosomes disrupted by the *Helicobacter pylori* VacA cytotoxin [72]. The mannose-6-phosphate receptor binds to **clusterin**. **PICALM** also binds to a nuclear exportin crm-1 (XPO1) used by the herpes simplex virus during its life cycle [48].

Gamma-adaptins (GGA, GGA2, GGA3) bind to clathrins and mannose-6-phosphate receptors and regulate protein traffic between the Golgi network and the lysosome and the sorting of mannose-6-phosphate receptors (IGF2R and M6PR) at the trans-Golgi network [73]. This network is also related to important Alzheimer's disease susceptibility genes as the interactions culled from NCBI gene show that GGA1 binds to the sortilin-related receptor, **SORL1,** and the **APP **cleaving beta-secretase **BACE2**, while GGA2 binds to the beta-secretases **BACE1** and **BACE2,** **SORL1** and the prolyl-isomerase **PIN1**.

**CD2AP**, primarily expressed in dendritic cells and B lymphoblasts http://www.biogps.org/#goto=genereport&id=23607/, is a scaffolding molecule that regulates the actin cytoskeleton and is primarily associated with the T-lymphocyte marker protein CD2. CD2 stimulates T cell activation and is involved in the creation of contacts between antigen presenting cells and T cells (the immunological synapse), effects mediated via **CD2AP** and clathrin [74]. **CD2AP** is also involved in the entry of the helicobacter vacuolating toxin VacA and connects the actin cytoskeleton to early endosomes containing VacA [75]. CD2 is cleaved by gingipain proteases from *P. gingivalis* [76].

**CD2AP** also binds to the actin-bonding protein, cortactin (CTTN), a protein that is exploited by several bacteria (*Escherichia coli, Shigella, Neisseria, Rickettsia, Chlamydia, Staphylococcus, Cryptosporidium, *and *Helicobacter pylori*), fungi (*Candida Albicans*), and viruses (Vaccinia) enabling them to modify the actin cytoskeleton, which they use for transport [77–79]. **CD2AP** has not been specifically associated with herpes simplex, although the actin cytoskeleton is exploited by this and many other viruses [80].

**CD2AP** also associated with E-Cadherin, (CDH1) [81]. The ectodomain of E-cadherin is involved in bacterial adherence to mammalian cells [82]. E-Cadherin binds to the *H. pylori* toxin CagA [83] and is also cleaved by the *Helicobacter pylori* protein HtRA allowing the pathogen to invade the intracellular compartment [84]. CDH1 and CDH5 expressions are increased by *C. pneumoniae* infection of human brain microvascular endothelial cells, contributing to vascular permeability changes and atherosclerosis [85].

Bridging integrator 1 (**BIN1**), also known as amphiphysin 2, is primarily expressed in the pineal and skeletal muscle, or otherwise ubiquitously http://www.biogps.org/#goto=genereport&id=274/. It is also involved in the clathrin-mediated endocytosis machinery [86] and binds to dynamins that regulate the clathrin network [87] including DNM1 and the herpes simplex binding partner **DNM2** [88] and to clathrins and the alpha adaptins, AP2A1 and AP2A2 [89]. **BIN1** also participates in phagocytosis in macrophages and is associated, but only transiently, with early phagosomes; however, it is retained on vacuoles containing *Chlamydia pneumoniae*, an effect that reduces the ability of the macrophage system to kill the bacteria via nitric oxide generation. Macrophages expressing a dominant negative **BIN1** internalise *C. pneumoniae*, but do not allow their killing [90]. BIN1 also binds to a number of alpha integrins (ITGA1, ITGA3, and ITGA6) [91]: integrins are used for attachment by many viruses, bacteria, and fungi and may serve as pattern recognition receptors regulating the immune response [92]. Individual integrins bind to many others, forming heteromeric complexes; for example, ITGA1 binds to ITGA3 or ITGA6, while ITGA3 binds to ITGB1 (a receptor for the *H. pylori* protein CagA [93]), ITGB4, or ITGB5, and ITGA6 binds to ITGB1 and ITGB4 (data from NCBI gene).

### 3.3. The Immune Network (APOE, BIN1, CD2AP, CD33, MS4A2) (Figure 4)

**CD33**, mainly expressed in myeloid cells, monocytes, and dendritic cells (http://www.biogps.org/#goto=genereport&id=945/), is a member of the sialic acid binding Immunoglobulin g-like lectin (SIGLEC) family. **CD33**-related SIGLECs regulate adaptive immune responses and are also important as macrophage pattern recognition receptors for sialylated pathogens, including enveloped viruses [94]. **CD33** binds to alpha2-3- or alpha2-6-linked sialic acids (N-acetyl neuraminic acid) [95]. These particular sialic acids are expressed on the surface envelope glycoproteins (B, D, and H) of the herpes simplex virion, and these residues are required for viral entry into cells [96]. N-acetyl neuraminic acid is expressed by *C. neoformans*, is involved in fungal adhesion to macrophages [97], and is also a component of the cell wall of *B. burgdorferi *[98], while *Helicobacter pylori* adhesins also bind to this particular form of sialic acid [99, 100] as does *P. gingivalis* [101].

**BIN1**, as well as its relationship to the clathrin mediated endocytosis machinery, also regulates the expression of indoleamine 2,3-dioxygenase (IDO1), an enzyme that catalyzes the first rate-limiting step in tryptophan metabolism to N-formyl-kynurenine [102]. IDO1 upregulation is an important defence mechanism against pathogenic bacteria, many of which are unable to synthesise tryptophan. Their survival is compromised by the diversion of tryptophan metabolism to kynurenines [103]. This IDO1 response is also deleterious to other pathogens and parasites, including *T. gondii*, and to a number of viruses, including herpes simplex and other herpes viruses [104]. IDO1 protein expression is localised to plaques and tangles in the Alzheimer's disease brain. IDO1 activation can lead to the production of toxic tryptophan derivatives such as 3-hydroxyanthranilic acid or the N-methyl-D aspartate receptor agonist and excitatory neurotoxin, quinolinic acid [105] (**GRIN2B, GRIN3A**). Plasma tryptophan levels are also lower in the ageing population and in Alzheimer's disease, a pattern accompanied by immune activation, and by increased concentrations of quinolinic acid [106, 107].

**MS4A2**, expressed mainly in the tonsils, lymph nodes, B cells, and dendritic cells http://www.biogps.org/#goto=genereport&id=931/, is a component of the immunoglobulin E (IgE) receptor, which is involved in allergic responses in which allergens bound to receptor bound IgE result in the activation of allergic mediators such as histamine [108]. Mice immunised with inactivated herpes simplex develop IgE-specific antibodies to the virus [109]. High levels of IgE are also observed in man following recurrent herpes simplex infection [110] and human IgE antibodies are also known to interact with herpes family viruses including HSV-1 and 2 and the Epstein-Barr and cytomegalovirus [111] and also to *C. pneumoniae*, *H. pylori*, and *B. burgdorferi *[112–115]. IgE-related allergic responses are also involved in *C. neoformans* infection [116]. Other members of this gene cluster (including **MS4A4A**,** MS4A4E**, and** MS4A6A)** are also structurally related to the immunoglobulin E receptor and to CD20 (MS4A1) and also regulate B cell and T cell proliferation and/or differentiation [117, 118].

**EPHA1** is an ephrin receptor, primarily expressed in the liver and otherwise ubiquitously (http://www.biogps.org/#goto=genereport&id=2041/). Only three protein/protein interactions for **EPHA1** are reported in the NCBI gene interaction section, including its ligand EFNA1, the anaplastic lymphoma receptor tyrosine kinase (ALK), and a SMAD-specific E3 ubiquitin protein ligase 2 (SMURF2). EFNA1 is one of several proteins identified as being important in the entry of *C. pneumoniae* into human coronary artery endothelial cells [57]. SMURF2 is known to bind to the VP22 tegument protein of herpes simplex [119] and plays a role in clathrin-mediated endocytosis and the subsequent ubiquitin-related proteasomal degradation of **TGF beta** receptors, to which it binds [120]. **Clusterin** is a ligand for **TGF beta** receptors (TGFBR1/TGFBR2) [121]. **TGF beta** signalling exerts immunosuppressive effects and inhibits host immunosurveillance and the recruitment of immunocompetent cells by chemokines [122]. ALK is ubiquitously expressed (http://www.biogps.org/#goto=genereport&id=238/). It plays a role in neural development, and its expression decreases with age [123]. ALK is best characterised via its relationship with lymphomas, caused by ALK gene fusion with any of several other housekeeping genes [124]. Its key involvement in lymphoma suggests a role in the immune network although the function of the normal ALK protein is poorly understood.

### 3.4. Lipoprotein Related (APOE, ABCA7, CLU) (Figures 2 and 5)

**ABCA7** is an ATP-binding cassette transporter, predominantly localised in the pineal gland and cells of the immune network (T cells, natural killer cells, and dendritic cells http://www.biogps.org/#goto=genereport&id=10347/). The lipoproteins **APOA1** and **APOE** are substrates for **ABCA7**, and in cultured HEK-293 cells, plasma membrane-situated **ABCA7** increases the efflux of phosphatidylcholine and sphingomyelin efflux to **APOA1** and **APOE**, with no effect on cholesterol efflux [125]. However, cholesterol efflux to lipid-laden **APOE**, but not to lipid free **APOE**, is increased by **ABCA7** expression in HEK-293 cells [126]. Sphingomyelin is enriched in extracellular herpes simplex viral membranes: this sphingomyelin, together with phosphatidylserine, is collected by the viral envelope during viral passage from the nuclear membrane to the exocytosis pathway [127]. Herpes viral infection leads to an increased incorporation of phosphate into membrane sphingomyelin of the host [128]. Inhibition of sphingomyelinase has also been shown to markedly reduce herpes simplex viral reproduction [129] and also inhibits the antifungal effects of neutrophils against *C. neoformans* infection. Sphingomyelin is a receptor for the Helicobacter toxin VacA [130] and is also incorporated into inclusion bodies in *C. pneumoniae*-infected cells [131]. Phosphatidylcholine plays an important role in the fusion of herpes simplex glycoproteins B and H with the host cell lipid membrane, a process used in viral entry [132]. Phosphatidylcholine is also able to trigger capsular enlargement in *C. neoformans* infection [133].

**ABCA7** expression increases the extracellular surface deposition of ceramide (derived from sphingomyelin) [134]. Ceramide, a potent activator of apoptosis, as well as its downstream target, caspase 3 (CASP3) are both able to reactivate the herpes simplex virus from latency [135]. Ceramide is also incorporated into *C. pneumoniae* inclusions, an effect that may play a role in the antiapoptotic effects of this bacterium [136]. **APOA1** exerts antiviral effects against herpes simplex and inhibits viral entry into cells as well as viral-induced cell fusion and intercellular spread [137]. In macrophages, **ABCA7** is expressed intracellularly and does not participate in cholesterol or phospholipid efflux, instead playing a role in the phagocytosis of apoptotic cells, an important general defence mechanism against invading pathogens [62, 138].

#### 3.4.1. Apolipoprotein E

Possession of the **APOE**4 allele facilitates the entry and transmission of herpes simplex in mice models [139]. In man, **APOE** is also involved in hepatitis C, HIV-1, and herpes simplex infectivity [140–143], and **APOE4** facilitates the binding of *C. pneumoniae* elementary bodies to host cells [144].

**APOE** mRNA is primarily expressed in the liver, adipocytes; kidney and brain, with very low expression in the peripheral immune network (http://www.biogps.org/#goto=genereport&id=348/) but nevertheless plays an important role in the immune system. For example, the presence of the **APOE**4 allele is associated with an enhanced macrophage inflammatory response, and cytokine responses to the intracerebral injection of lipopolysaccharide are increased in **APOE**4 transgenic mice, which also exhibit increased microglial activation. The anti-inflammatory effects of 17-beta-oeastradiol on microglia are also reduced in such animals [145, 146]. C-reactive protein (**CRP**) levels are also decreased in **APOE**4 carriers [145–147]. **CRP** is an acute phase protein that binds to phosphocholine on dead or dying cells and on bacteria, subsequently activating the complement pathway [148]. Resistance to infection (*Klebsiella pneumoniae)* or endotoxaemia is also decreased in **APOE** knockout mice [149].

In addition, atherosclerosis is induced or worsened by infection with a number of relevant pathogens (Cytomegalovirus, herpes simplex, *Helicobacter pylori*, influenza, *C. pneumoniae* or *P. gingivalis*) in **APOE** knockout mice [150–156]. *Helicobacter pylori* is able to promote atherosclerosis in heterozygous **APOE** (+/−) LDLR (+/−) mice, which is associated with an immune response to the bacterial heat shock protein hsp60 [157]. 

### 3.5. Other GWAS Genes (Figure 4)

Prior to the very large GWAS collaboration, several other genes had been identified in smaller genomewide studies (**APOC1, CELF2, DISC1, FAM113B, GAB2, MTHFD1L, PAX2, PCDH11X, PVRL2 RFC3, SASH1, TOMM40, TTLL7, and ZNF224**). **PVRL2** is a receptor for herpes simplex (HSV-1 and HSV-2) [158], and the mitochondrial translocator, **TOMM40**, a receptor for certain chlamydial species [159]. The replication factor **RFC3 **is part of a complex necessary for human DNA polymerase activity, a process exploited by many viruses including herpes simplex, whose virion component ICP34.5 binds to proliferating cell nuclear antigen (PCNA), an **RFC3** binding partner and also a cofactor for DNA polymerase [160]. **ZNF224** is a transcriptional repressor binding to the protein arginine methyltransferase, PRMT5 [161]. Protein arginine methylation is important in viral infection and replication, as well as in cytokine signalling, and a related arginine methyltransferase, PRMT1, regulates herpes simplex replication via methylation of the ICP27 viral gene [162].

**DISC1** is a component of the microtubule-associated dynein motor complex used in viral traffic [163]; **TTLL7** (tubulin tyrosine ligase-like family, member 7) also regulates tubulin phosphorylation [164] and can again be related to viral traffic along the microtubule network (see below). **CELF2** (also known as CUGBP2) is a member of the APOBEC1 cytidine deaminase mRNA editing complex that also controls herpes simplex viral replication [165]. **GAB2 **is a member of the GRB2-associated binding protein family which act as adapter hubs transmitting signalling via cytokine and growth factor receptors, and T- and B-cell antigen receptors (definition from NCBI gene), while **PAX2** inhibits the expression of the antimicrobial peptide beta defensin (DEFB1) [166], a gene associated with HSV-1 and cytomegalovirus seropositivity in children with acute lymphoblastic leukaemia [167], as well as with *H. pylori* or chlamydial infections [168, 169], also endowed with antimicrobial activity against *C. neoformans* and other pathogens [170]. **MTHD1L** (methylenetetrahydrofolate dehydrogenase (NADP+ dependent) 1-like) is involved in the mitochondrial synthesis of tetrahydrofolate which in turn is important in the de novo synthesis of purines and thymidylate and in the regeneration of methionine from homocysteine (definition from NCBI gene). Many pathogens, including herpes simplex, express thymidylate kinases, which are important for viral replication and a target for acyclovir [171]. Hyperhomocysteinaemia correlates with *C. pneumoniae* IgG immunoreactivity in carotid artery atherosclerosis [172] and is also associated with *H. pylori* infection in the context of atherosclerosis [173]. The apolipoprotein **APOC1** is a component of high-density lipoprotein: herpes simplex is present in all lipoprotein blood fractions in blood (VLDL, LDL and HDL) and the lipid component of these lipoproteins binds to viral glycoprotein B [174] (c.f. **APOA1, APOA4, APOA5, APOC1, APOC2, APOC3, APOC4, APOD, **and** APOE)**. No immediately apparent pathogen-relevant interactions were found for **FAM113B** (expressed exclusively in T cells, dendritic cells, and natural killer cells http://www.biogps.org/#goto=genereport&id=91523/), **PCDH11X **(which is ubiquitously expressed http://www.biogps.org/#goto=genereport&id=27328/), or **SASH1** (primarily expressed in the brain and lung although also in other tissues, including the immune network http://www.biogps.org/#goto=genereport&id=23328/) although the pathogen/immune theme is clearly carried through, particularly in relation to herpes simplex, in this second rank of Alzheimer's disease susceptibility genes.

### 3.6. Beta Amyloid Processing (Figure 2)

**APOE**, **clusterin**, and **complement receptor 1** play key roles in beta amyloid clearance as do two further herpes simplex binding proteins **APOA1**, and alpha-2 macroglobulin (**A2M**). This is primarily mediated via lipoprotein receptors. A2M, or **APOE**-bound A*β*, is cleared by the lipoprotein receptor **LRP1**, while **LRP2** (megalin) clears clusterin-bound A*β*. **LRP8** is a receptor for both **APOE** and **clusterin**. **APOA1** is also involved in beta-amyloid clearance via its transporter **ABCA1**. The role of **ABCA7** has not been examined, although **APOA1** is also a ligand for this transporter (see above). The Varicella Zoster and herpes simplex glycoprotein E binding protein, **insulin-degrading enzyme**, are also involved in beta-amyloid degradation, as is caspase-3 which is activated by the herpes simplex viral US3 kinase. The HSV-1 binding protein, complement C3 is also a ligand for **LRP1** and **LRP8**, both of which play a role in C3 cellular uptake. Beta amyloid in the bloodstream is processed by its binding to complement C3, which subsequently binds to **complement receptor 1** on erythrocytes. The effects above are referenced in a recent review [48].

Clathrin-dependent endocytosis is also involved in the internalisation and recycling of neuronal **APP**, a procedure necessary for the subsequent cleavage of **APP **and the generation of beta-amyloid [175, 176], and in the neuronal [177], but not the microglial uptake of both soluble and aggregated beta-amyloids, the latter representing an important rout of disposal [178]. However, while knockdown of the clathrin assembly protein AP180 in a neuronal cell line does reduce beta-amyloid generation, **PICALM** knockdown does not [179]. The accumulation of beta-amyloid in the brain interstitial space, related to the prior endocytosis of **APP**, is clathrin dependent [180]. Clathrin-mediated endocytosis is relevant to many receptors, including members of the lipoprotein family (**LRP1, LRP2, LRP8, LDLR, VLDLR**) [181], all of which are involved in beta-amyloid clearance, as well as in cholesterol and lipoprotein physiology [182].

**ABCA7** plays a role in beta-amyloid secretion, which is increased in Chinese hamster ovary cells expressing APP and **ABCA7**. This was related to an effect on **APP** intracellular retention, rather than on secretase-mediated proteolysis of **APP** [126].

**Gamma-secretase** cleaves **APP**, and other gamma-secretase substrates also play key roles in **APP** processing (ADAM10) [183], lipid and cholesterol function (**LRP1, LDLR, VLDLR**), and other processes relevant to Alzheimer's disease, for example, NOTCH signalling [184].

No other immediately apparent relationships with beta-amyloid could be found by literature survey for **CD2AP**, **CD33**, and **EPHA1** or for **MS4A**-related proteins, although such are not precluded.

#### 3.6.1. The Microtubule Network and Tau Phosphorylation

Many pathogens, including herpes simplex, *helicobacter, chlamydiae*, *P. gingivalis*, and *C. neoformans* [185–188], use the microtubule network that serves as a useful railway track between various cellular compartments, and may hijack dynein and kinesin motors for this purpose (see http://www.polygenicpathways.co.uk/herpeshost.html for herpes simplex). **Tau** (**MAPT**) stabilises microtubules by interacting with tubulins and promoting microtubule assembly [189]. When **tau** is phosphorylated, by any of several kinases, microtubules become disorganised. **Tau** hyperphosphorylation and neurofibrillary tangles are among the core pathologies of Alzheimer's disease [190] and can be promoted by herpes simplex infection [191]. In relation to viral/human protein homology, herpes simplex proteins are homologous to a number of kinases known to phosphorylate **tau** (GSK3A, **GSK3B**, MAPK1, and CAMK2B) suggesting that **tau** phosphorylation could be a direct result of a viral kinase [192].

#### 3.6.2. APP and Gamma Secretase

**APP** plays a key role in the herpes simplex life cycle and is involved in its intracellular transport [193], an effect likely related to the ability of both APP and the herpes simplex protein, US11, to bind to the APP and kinesin binding protein APPBP2 (also known as pat1) [194, 195].

**The AntiMicrobial Effects of Beta-Amyloid**

Beta-amyloid is an antimicrobial peptide with broad spectrum activity against a variety of yeasts and bacteria, effects that were attenuated by anti-A*β* antibodies [196], Beta-amyloid also has antiviral effects and, like acyclovir, attenuates the stimulatory effects of herpes simplex on miRNA-146a levels in neuronal cells [197]. Beta-amyloid also activates innate immune responses via the activation of pattern recognition receptors, such as Toll receptors (**TLR2, TLR4**), which are also involved in beta-amyloid clearance [198, 199]. The antimicrobial, antiviral, and immunostimulant properties of beta amyloid are, however, likely to be abrogated by the presence of beta-amyloid autoantibodies in the sera of the ageing population and in Alzheimer's disease [200]. As immunogenic regions of beta-amyloid are homologous to similar regions within proteins expressed by all of the principal pathogens discussed in this paper, such antibodies are likely to be derived from antibodies raised to numerous pathogens (see below).

**Gamma Secretase: Localisation to Dendritic Cells and Cleavage of Pathogen Receptors**

**Gamma secretase** is constituted of four components: the presenilins (**PSEN1** or **PSEN2**), anterior pharynx-defective-1 (**APH1A**), the Presenilin enhancer-2 (**PSENEN**), and nicastrin (**NCSTN**) [201]. While all components are expressed in cerebral tissue, the major focus of distribution is within cells of the immune network; dendritic cells, myeloid cells, and monocytes for **PSEN1** http://www.biogps.org/#goto=genereport&id=5663/; dendritic cells and natural killer cells for **PSENEN** http://www.biogps.org/#goto=genereport&id=55851/, dendritic cells and myeloid cells for nicastrin http://www.biogps.org/#goto=genereport&id=23385/ and B cells, dendritic cells, natural killer cells, and myeloid cells for **APH1A**. The substrate, **APP**, is the only gene in this set that appears to be preferentially distributed in brain compartments, but, as **with gamma-secretase** components, it is also highly expressed in dendritic cells of the immune system (http://www.biogps.org/#goto=genereport&id=351/). The primary function of such cells is to process antigens and present them to B cells and T cells. They scout for and recognise pathogens via the agency of numerous pattern recognition receptors, for example, Toll receptors (**TLR2, TLR4**), or viral DNA sensors, expressed on their surface [202, 203].

As well as cleaving **APP**, **gamma secretase** is involved in the proteolysis of at least three herpes simplex receptors, nectin 1 alpha (PVRL1) [204], and syndecans (SDC1, SDC2) [205]. SDC1 is also a receptor for HIV-1, Hepatitis E, and the human papillomavirus [206–209], while SDC3, also a **gamma secretase** substrate, is an HIV-1 and papillomavirus receptor [210, 211].

Several other **gamma secretase** substrates (reviewed by Lleó and Saura [201]) also function as viral/pathogen receptors, including ADAM10, a receptor for the cytotoxin Staphylococcus aureus alpha-haemolysin [212], CD44, an entry receptor for *C. neoformans* [213], CD46, a receptor for adenoviruses, measles virus, human herpes virus 6 (HHV-6), Streptococci, and Neisseria [214]: CD46 is also cleaved by a protease secreted by *P. gingivalis* [215]. Desmoglein-2 is an adenovirus receptor [216], while rhinovirus receptors include the lipoprotein receptors **LRP1**, **LDLR**, and **VLDLR** [217, 218]. **LDLR **is also a hepatitis C receptor [219]. NOTCH1 and NOTCH4 are activated by the Epstein-Barr virus [220, 221], while ERBB4 is a receptor for vaccinia and other pox viruses [222]. The low affinity nerve growth factor (NGFR) is a rabies virus receptor, [223], Ephrin B2, (EFNB2) a Nipah virus and Hendra virus receptor [224] and sialophorin (SPN), a receptor for the influenza A, and both human and simian immunodeficiency viruses [225, 226] and for the *C. neoformans* virulence factor, galactoxylomannan [227]. Fractalkine (CX3CL1) binds to the cytomegalovirus chemokine receptor, US28 [228], while the chemokine CXCL16 is a scavenger receptor for phosphatidylserine and oxidized low density lipoprotein [229]: phosphatidylserine is the major lipid membrane component in most bacteria [230]. Dystroglycan is a receptor for the Lymphocytic choriomeningitis and Lassa fever viruses [231, 232] and also for *mycobacterium leprae* (the Leprosy pathogen) [233].

The antimicrobial and immunostimulant effects of beta-amyloid, the cleavage of a number of pathogen receptors by **gamma secretase**, and the concentration of both **APP** and **gamma-secretase** components in dendritic cells suggest that a major function of this key group, implicated in both familial and late onset Alzheimer's disease, is dedicated to pathogen defence, and that increased beta-amyloid generation is primarily a defence mechanism to rid the body (and brain) of invading pathogens: This scenario is supported by the ability of herpes simplex, *C. pneumoniae*, and *B. burgdorferi *to increase beta-amyloid deposition [234–236]. One might expect many other pathogens to increase beta-amyloid deposition, and that, as has been noted in atherosclerosis, (a component of Alzheimer's disease pathology), the final extent of risk may depend upon the overall pathogen burden, rather than upon any specific pathogen [237].

#### 3.6.3. Autoantibodies Derived from Pathogens as Contributory Causative Agents

Viruses and bacteria express proteins containing short contiguous amino acid stretches (pentapeptides or more, or longer gapped consensi) that are identical to those in human proteins: these pathogen/human consensi number in millions and concern all human proteins [238–241].

Autoantibodies, which are observed in many, if not most human diseases, are often regarded as an epiphenomenon of little consequence. However, they can traverse the blood brain barrier [242] (which is compromised in Alzheimer's disease [243]) and are also able to enter cells, essentially by hitching a ride on viruses, via high affinity IgG receptors (Fc gamma receptors) (**FCER1G**) in the case of the rhinovirus, or the SARS coronavirus, or via the tripartite motif protein, TRIM21, in the case of adenoviruses, where they are able to activate an intracellular immune attack. It would appear that the cellular entry of antibody laden viruses is diverted from their usually preferred receptors towards those used by antibodies [244–246]. This may be relevant to the **MS4A** family. Fc gamma receptors are localised in microglia and astrocytes in the brain and their expression is upregulated by blood brain barrier disruption [247], while TRIM21 appears to be exclusively localised in peripheral immunocompetent cells (http://www.biogps.org/#goto=genereport&id=6737/).

This ability places autoantibodies in a rather more sinister context, as their targeting of extracellular and intracellular human proteins would be expected to effect protein knockdown, a strictly immunopharmacological effect, as well as immune attack.

In multiple sclerosis, schizophrenia, and cystic fibrosis, as well as in Alzheimer's disease, numerous autoantigens targeted by the autoantibodies reported in these conditions, contain peptide sequences identical to those in the pathogens also implicated in the disease. Such regions of homology are focalised within epitope regions of the human autoantigen [192, 248–250]. In Parkinson's disease, antibodies to the Epstein-Barr virus, which has been implicated in postencephalitic adult and juvenile Parkinsonism, are also known to cross-react with synuclein, a key protein involved in neurodegeneration in this disorder [251–253]. In addition, 22 autoantigens reported in HIV-1/AIDS contain HIV-1/human matching sequences [254], supporting the contention that autoantibodies are in many cases antibodies initially raised to pathogens, which because of this homology, then target their human homologues. It has been argued that slightly dissimilar, rather than exact matches, are the more malignant in terms of autoimmunity, being less likely to be regarded as self, while the antibodies would retain low affinity for human counterparts [254, 255].

Autoantibody production would also be sustained, even after pathogen elimination, by continued encounter of the human homologue. The production of autoantibodies must be dependent upon the extent of pathogen/human matching, and thus by genes which determine human protein sequences. These pathogen/human matches are also highly and significantly enriched in the products of susceptibility genes implicated in Alzheimer's disease, multiple sclerosis, and schizophrenia [192, 248, 250]. Many genes related to Alzheimer's disease, including those described above, are involved in the immune network [10, 256], and the propensity for developing autoantibodies to particular proteins is also genetically determined and inherited [257]. Thus, despite the fact that all human proteins likely possess pathogen homologues, whether or not autoantibodies will be produced will depend on the extent of human/pathogen matching (determined by human genes and the strain of pathogen encountered), on whether the pathogen protein is deemed as self or nonself (a factor determined soon after birth) and on other genetic factors related to the immune network, and autoimmunity. Somatic hypermutation, that drives the creation of multiple antibodies and which selects against those reacting to self, is disrupted in autoimmune disorders [258]. These links suggest an interplay, applicable to many diseases, where susceptibility gene products, risk promoting pathogens and autoimmunity can all be related via protein sequence homology.

It has also been noted that autoantigens have a tendency to relate to proteins known to bind to dermatan sulphate, a component of dead cells [259] and a constituent of glycosaminoglycan receptors for many bacteria and viruses [260].

#### 3.6.4. Sequence Comparisons: Beta-Amyloid, NGF, and Tau versus Pathogen Proteins

All three of these proteins are autoantigens in Alzheimer's disease and were chosen for analysis because of the ability of their antibodies to promote features of Alzheimer's disease, *in vivo*. In mice, immunisation with neuronal ***tau*** produces neurofibrillary tangle-like structures, axonal damage, and gliosis, as in Alzheimer's disease [261]. In addition, in transgenic mice, initially expressing NGF antibodies only in lymphocytes, NGF antibodies subsequently enter the brain provoking extensive cortical degeneration, cholinergic neuronal loss, **tau** hyperphosphorylation, and beta-amyloid deposition [262]. Beta-amyloid autoantibodies are also able to promote meningoencephalitis, both in laboratory models and in clinical trials [263, 264]. Beta-amyloid plaques contain numerous inflammatory proteins, and even without meningoencephalitis, these are commonly found within the walls of meningeal and medium-sized cortical arteries in Alzheimer's disease [265].

Almost the entire length of the **tau** protein (638/776 amino acids = 82.2%) was predicted as immunogenic (B cell epitope), as defined by the server set cutoff index of 0.35. For the analysis in Table 1, only regions of the **tau** protein with an immunogenicity index >2.5 were examined for homology. For other proteins (NGF and beta-amyloid), the analysis concerned immunogenic regions above the cutoff value of 0.35.

All pathogens express proteins with homology to each autoantigen, specifically within predicted B-cell epitope regions of the human autoantigen (Table 1), suggesting that antibodies raised to any could be responsible for targeting these human proteins, under the appropriate circumstances. Perversely, the successful elimination of the pathogen via antibody production could set in motion the very autoimmune responses that may be crucial to the development of Alzheimer's disease, a Pyrrhic victory, which would also be promulgated by any further encounter with these very common pathogens, or by structurally related proteins from other pathogens, as well as by continual encounter of the human autoantigen homologue (see Section 3.6.5).

As well as autoantibodies to these three proteins, a number of autoantibodies targeting highly relevant proteins have been reported in Alzheimer's disease. These have functional effects on their target proteins and include antibodies that block the activity of ATP synthase, induce apoptosis, and increase the cellular uptake of high density lipoprotein [266, 267], antibodies to cholinergic neurones that cause immunalysis of brain synaptosomes [268], antibrain antibodies that enhance intraneuronal beta-amyloid deposition [269] as well as antibodies to the nicotinic receptor **CHRNA7**, that displace its ligand alpha-bungarotoxin [270]: autoantibodies to the receptor for advanced glycation products (**AGER**) [271], to the antimicrobial peptide **S100B**, have also been reported [272].

#### 3.6.5. Population Genetics: Susceptibility Genes Related to the Cause of the Disease, rather than to the Disease Itself

Using a classical example from the field of population genetics and Darwinism, the light coloured genes of the peppered moth favour its selective predation by *many different* birds when it alights on dark trees covered with soot pollution, while darker melanised forms are selectively targeted on lighter coloured trees [273]. The coloration susceptibility genes, or the variety of tree (risk factors), do not kill the moth but allow *several *causes to do so. The causes can hide in plain sight, as epidemiological studies, as applied to human diseases, could conclude that the birds are not killing the moths, as they are always present, on both sets of trees, in both genetic conditions, whether the moths are alive or dead (c.f. the ubiquitous *C. neoformans* and many other common pathogens). Many of the pathogens implicated in Alzheimer's disease (herpes simplex*, Borrelia burgdorferi* and *C. pneumoniae*), and several other risk factors (cholesterol, homocysteine, diabetes, or vitamin A or nerve growth factor deficiency) are able to promote cerebral beta-amyloid deposition in animal models, without the aid of any gene variant. Subsets of susceptibility genes can be related to each of these amyloidogenic pathways [1]. In the case of genomewide association studies, the genes returned, as well as **APP**, beta amyloid, and **gamma secretase**, seem intimately concerned with pathogen life cycles and defence and the immune network. This suggests that the diverse microbial risk factors as well as other dietary and environmental factors implicated in Alzheimer's disease are in fact causative agents, whose deleterious effects are conditioned by susceptibility genes. As the environmental risk factors are amenable to therapy, while the susceptibility gene products have so far proved largely resistant, this suggests numerous ways with which to tackle the problem of Alzheimer's disease.

**Herpes Simplex Reactivation**

Alzheimer's disease plaques and tangles are highly enriched in human proteins used the herpes simplex during its life cycle, as well as in many immune-related proteins, suggesting that immune attack on a reactivated virus, diverted to neurones, which contain the complement membrane attack complex, may be ultimately responsible for the extensive neuronal destruction seen in Alzheimer's disease [15]. The herpes simplex virus establishes latency in neurones, existing as a dormant form where only the latency transcript is expressed. A number of factors again related to susceptibility genes and environmental risk factors in Alzheimer's disease can be related to herpes simplex latency and reactivation. The cerebral herpes simplex viral load is decreased in **APOE** knockout mice, while the viral load is much increased in **APOE4 **transgenic mice, compared to APOE3 mice, suggesting that **APOE4 **favours the establishment of a greater number of latent viruses in cerebral tissue [274]. In neuroblastoma cells, this latent form may even exert beneficial effects, blocking apoptosis and promoting neurite extension via AKT1 upregulation [275]. However, this latent form can be reactivated by NGF deprivation [276], and NGF promotes viral latency via the TrkA receptor **NTRK1 **[277], the expression of which is reduced in the Alzheimer's disease brain [278]. (Relevant pathways and genes: neurotrophin signalling (**GSK3B, NTRK1, NTRK2, PIK3R1, and SOS2**). Vitamin A supplementation in rats increases the cerebral levels of both NGF and **BDNF** [279], while oestrogen deficiency lowers cerebral NGF levels, an effect reversed by 17-beta oestradiol [280], which is, however, also able to reactivate the virus, via oestrogen receptor alpha (**ESR1**) [281]. High levels of total oestradiol have been reported as a risk factor for Alzheimer's disease in both women and men [282, 283]. Vitamin A related gens include **APOE4**, the isoform least able to transport the vitamin A precursor retinyl palmitate, **A2M ABCA1 ALB ALDH2 APOA1 CHD4 CLU CYP46A1 ESR1 GSTM1 GSTP1 HSPG2 KLF5 LIPA LPL LRAT LRP2 LRPAP1 MEF2A NPAS2 NR1H2 PARP1 PIN1 POU2F1 PPARA PPARG RXRA THRA TTR UBQLN1 VDR** and many others controlled by retinoid receptor element (reviewed in [126]).

The virus can also be reactivated by heat [284], **IL6 **[285], or **TNF **[286]. **IL6** plasma and CSF levels have been reported to be increased in Alzheimer's disease and the secretion of **IL6** from monocytes is increased [287–289]. **IL6** plasma levels are raised by infection with *C. pneumoniae* [290] or *Helicobacter pylori* [291], and **IL6** production in monocytes is stimulated by *C. neoformans* [292]. Stress-activated corticosterone is also able to reactivate the virus [293] (pathway = steroid hormone biosynthesis: **COMT, CYP19A1, HSD11B1**). Cortisol levels are increased in the ageing population and in Alzheimer's disease [294, 295]. A number of related stressors including adrenaline [296], or downstream effectors such as cyclic AMP, protein kinase A, or C activation [297] are also able to reactivate the virus. Herpes simplex reactivation is blocked by beta-receptor antagonism (**ADRB1**) [296, 298]. Glucocorticoids and prostaglandins are also able to reactivate the virus [299]: cyclooxygenase inhibitors (**PTGS2**), celecoxib and indomethacin block viral reactivation and nonsteroidal anti-inflammatory use has been associated with a lower incidence of Alzheimer's disease. Lysophosphatidic acid is also able to reactivate the virus [300] (**LPAR5**). Hypoxia also increases the replication of herpes simplex [301]: transient cerebral ischaemia also lowers NGF levels [302], and these effects are relevant to the cerebral hypoperfusion seen in ageing and in Alzheimer's disease [303]. Vitamin A and the retinoic acid isomers all-trans-, 9-cis-, and 13-cis-Retinoic Acid are all able to reduce viral replication [304]. Low Vitamin A levels are a problem in the ageing population and even in successfully ageing persons can be observed in 50% of the population over the age of 80–85 [305]. Low vitamin A levels are also a risk factor for Alzheimer's disease [306]. Mice deficient in the nitric oxide synthase **NOS2** are also more susceptible to herpes virus infection, and reactivation occurs is stimulated by caspase 3 activation (CASP3) [135].

Thus, fever or cytokine release induced by diverse infections might be expected to reactivate herpes simplex as would several of the conditions associated with Alzheimer's disease (cerebral hypoperfusion, low cerebral NGF levels, vitamin A deficiency, or high oestradiol levels).

#### 3.6.6. The Microbiome in Alzheimer's Disease

These pathogens form a very small proportion of an extensive human microbiome comprising of trillions of bacteria, viruses, and other pathogens, whose influence on diseases is increasingly recognised [307]. Individual species may exert benevolent or malevolent effects which may well be dictated by the very extensive sequence homology between human and pathogen proteins which enables pathogens to intercalate with numerous important signalling networks, via competition with their human counterparts [239, 241, 248, 250, 254]. These networks are implicated in diseases, and their efficiency, as well as pathogen/host homology, is dictated by susceptibility genes. This host/pathogen matching covers the entire human proteome and is represented by millions of short consensi of pentapeptides or more, not counting the longer gapped consensi. Clearly, powerful algorithms are needed to trawl human and pathogen proteomes and to link these homologies to susceptibility genes and to epitopes and autoantigens.

The principles discussed here may apply to many other, if not most, human diseases.

**Protective Agents in Alzheimer's Disease**

Statins [308], fish consumption [309], omega-3 polyunsaturated fatty acids [310], the Mediterranean diet [311], nonsteroidal anti-inflammatories [312], or the generally healthy life-style of nuns living in convents [313] have all been associated with a reduced incidence or severity of Alzheimer's disease, while homocysteine lowering B vitamins and folate have been shown to slow the rate of brain atrophy in cognitively impaired elderly patients [314]. A rather pessimistic study [315] recently stated that no firm conclusions could be drawn on the association of any modifiable factors with risk of Alzheimer's disease. While this may be a justifiable statistical conclusion from meta-analysis, this is not to reckon with the diversity of the underlying genomic platform of each individual or with the profusion of diverse interacting risk and protective factors (epistasis, gene/environment, and environment/environment interactions).

As with the risk factors, protective agents can be linked to genetic and pathological pathways involved in cholesterol and lipid function (statins, fish, and diet, e.g., **ABCA1, ABCA2, ABCA7, ABCG1, ACADS, ALDH2, APP, APOA1, APOA4, APOA5, APOC1-4, APOE, CH25H, CYP46A1, DHCR24, FDPS, HMGCR, HMGCS2, HSD11B1, LIPC, LRP1, LRP2, LRP8, LDLR, LIPA, LPL, OLR1, PPARA, PPARG, PTPLA, VLDLR, NPC1, NPC2, SOAT1, SREBF1**), homocysteine, methionine, and folate metabolism (folate and vitamins, e.g., **BLMH, CBS, COMT, NAT2, MTHFD1L, MTHFR,MTR, MTRR, PON1, PON2, PON3, VDR**) and inflammatory pathways (NSAID'S e.g., **C4A, C4B, CD2AP, CD33, CFH, CCL2, CCL3, CCR2, CSF1, CLU, CR1, FAS, GSK3B, IL1A, IL1B, IL6, IL8, IL10, IL18, PLAU, SERPINA1, SERPINE1, SERPINF2, PTGS2, TGFB1, TNF**). As with the risk factors, the success of such protective agents is likely to be determined by genes and other confounding factors. In this genomic era, affordable whole genome sequencing will soon be achieved, ushering in an age of more effective treatments tailored to individual genetic profiles. Alzheimer's disease is clearly multifactorial with many related genetic and environmental risk factors, several underlying pathologies, and several available protective strategies. A recent study has also shown that many other seemingly benign factors (eyesight, hearing, denture wearing, stomach, kidney, bladder or bowel problems, coughs, and colds) as well as high blood pressure and diabetes, constituting a frailty index, combine to markedly increase both the incidence and severity of Alzheimer's disease [316]. A further study, identifying physical inactivity, depression, smoking, mid-life hypertension or obesity, low education, and diabetes as key risk factors, has estimated that ~50% of Alzheimer's disease cases may be preventable [317].

Elimination of the risk factors, including the regular detection and elimination of pathogens in the elderly, adherence to sensible dietary and vitamin supplement recommendations, and the genetically tailored use of certain drug regimens are together likely to be able to markedly reduce the incidence of Alzheimer's disease. Using phage display, it is now possible to express peptide fragments of the entire human proteome in a phage library, and to use this to trap autoantibodies in blood or other bodily fluid samples. The antigen expressed by the labelled phage can be identified by high throughput sequencing [318]. Such technology is likely to be extremely useful in characterising biomarkers and pathological immune processes as well as potential pathogen/human cross-reactivity.

Proteome-wide characterisation of the autoantibodies relevant to Alzheimer's disease (a move from GWAS to PWAS) would also be very informative as selective autoantibody removal via affinity dialysis might well be expected to influence the severity and progression of Alzheimer's disease.

Since the submission of this paper, Miklossy has reported a highly significant association between spirochete infection and Alzheimer's disease. As well as *B. burgdorferi*, several periodontal pathogen treponemas species were detected in brain samples and the pathological features of Alzheimer's disease were reproduced by infection *in vitro* [319]. In addition, two groups have reported a very extensive repertoire of autoantigens in Alzheimer's disease, which can be characterised with a high degree of accuracy by a definitive immunosignature [320, 321]. The principles outlined above could thus be tested by analysis of pathogen/autoantigen cross-reactivity.

## Figures and Tables

**Figure 2:**
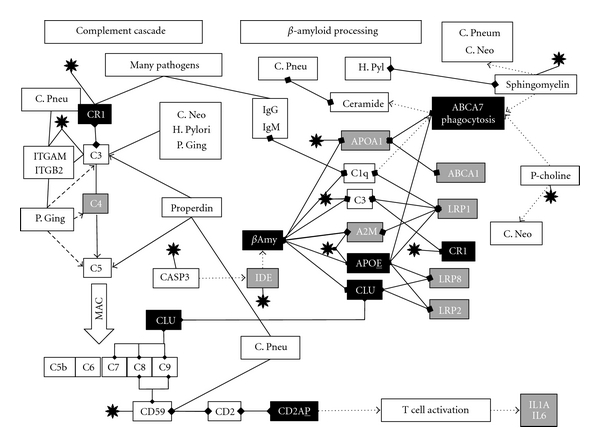
Portions of the complement cascade in relation to beta-amyloid processing: Alzheimer's disease susceptibility genes returned from very large genomewide association studies are in black, and those from the pre-GWAS era in grey. Binding interactions are indicated by linked diamonds and other effects by arrows. C. Neo: *Cryptococcus neoformans*; C. Pneu: *chlamydia pneumoniae*; H. Pyl: *Helicobacter pylori*; P. Ging: *Porphyromonas gingivalis*; C1–C9: complement components. MAC: membrane attack complex. P-choline: phosphatidylcholine; black star: Herpes simplex: see text for details.

**Figure 3:**
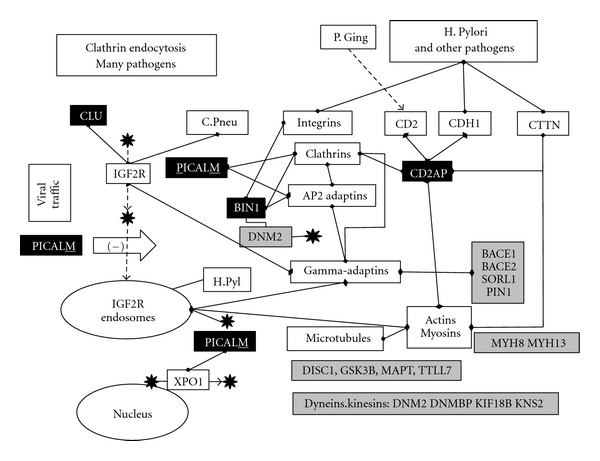
A schematic representation of clathrin-mediated endocytosis and intracellular transport pathways. Alzheimer's disease susceptibility genes returned from very large genomewide association studies are in black, and those from the pre-GWAS era in grey. Binding interactions are indicated by linked diamonds and other effects by arrows. C. Pneu: *chlamydia pneumoniae*; H. Pyl: *Helicobacter pylori*; P. Ging: *Porphyromonas gingivalis*; black star: Herpes simplex: see text for details.

**Figure 4:**
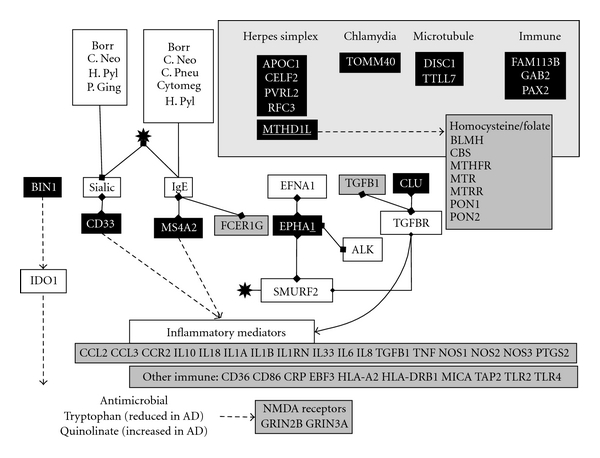
Immune-related genes: Alzheimer's disease susceptibility genes returned from very large genomewide association studies are in black, and those from the pre-GWAS era in grey. Binding interactions are indicated by linked diamonds and other effects by arrows. Borr: *Borrelia burgdorferi*; C. Pneu: *chlamydia pneumoniae*; Cytomeg: *cytomegalovirus*; H. Pyl: *Helicobacter pylori*; P. Ging: *Porphyromonas gingivalis*; IgE: immunoglobulin E, Sialic: alpha2-3- or alpha2-6-linked sialic acids: the genes in the top right square were returned from GWAS prior to the very large studies. black star: Herpes simplex: see text for details.

**Figure 5:**
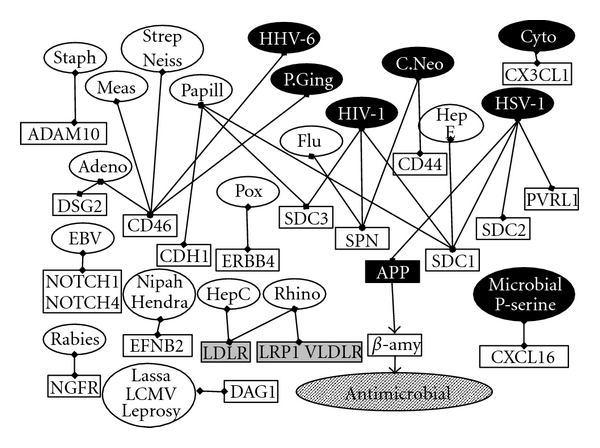
Gamma-secretase-mediated cleavage of viral and pathogen receptors. **Gamma secretase** substrates are indicated by the square boxes and their pathogen ligands in the oval boxes. Cyto: cytomegalovirus; C. Neo: *Cryptococcus neoformans*; Adeno: adenovirus; EBV: Epstein-Barr virus; Flu-influenza A.; HepC: hepatitis C; HepE: hepatitis E; HHV-6: human herpesvirus 6; HIV-1: human immunodeficiency virus; HSV-1: herpes simplex; Lassa: Lassa fever virus; LCMV: Lymphocytic choriomeningitis virus; Leprosy: *mycobacterium leprae*; Meas: measles; Neiss: *Neisseria*; Papill: papillomavirus; P. Ging: *Porphyromonas gingivalis*; Pox: Vaccinia and other pox viruses, Rhino: rhinoviruses; Staph: *Staphylococcus aureus*,Strep: *streptococcus*; P serine: phosphatidylserine: Alzheimer's disease susceptibility genes returned from very large genomewide association studies, and APP, are in black, as are the pathogens implicated in Alzheimer's disease Genes from the pre-GWAS era are in grey.

**Table 1a:** (a)

Position B-Amy	Amino acid	B-cell index	Alignments
1	D	*0.41*	*C. neoformans* +AE HDSG+ Borrelia burgdorferi DAE F H+SG EV *H. pylori* DA FRH HSV-1 +AE RH HHV-6 D FR DS *P. gingivalis* +AEFR *C. pneumoniae* DA EFRHD and +AEFR +SG
2	A	*0.35*	*C. neoformans* AEFR D GY+V *H. pylori* AEF D S YE and AE+RH+ Borrelia burgdorferi AEF H+ Cytomagalovirus AEFR HD HSV-1 AE R SG HHV-6 AE+ HD *P. gingivalis* A+F H+S and AEFR *C. pneumoniae* AEF DSG
3	E	*0.62*	*H. pylori* EFRHD HHV-6 EF DSG Borrelia Burgdorferi EFR DS *C. neoformans* EF R DS YE *P. gingivalis* E R DSGY V *C. pneumoniae* EF SGYEV
4	F	*0.73*	*C. neoformans* FRHDS Borrelia burgdorferi +RH SGY++ and F H+SG *H. pylori* F HD EV Cytomegalovirus FR SGY *P. gingivalis* +RHDS C. Pneumonia F H+SGY
5	R	*0.85*	*C. neoformans* R D GYEV *H. pylori* RHDS Y V and R SGYE Borrelia burgdorferi RH+ GY Cytomegalovirus RHD YE and RHDSG HSV-1 RH SG HHV-6 RHDS *P. gingivalis* R+DS Y+
6	H	*0.57*	*C. neoformans* HDSGY *H. pylori H. pylori* +DSGY and HD G EV and HD EV Borrelia burgdorferi H+SG Y+V HSV-1 HDSG *P. gingivalis* HDSG *C. pneumoniae* ++SGY+V
7	D	*0.69*	*C. neoformans* DSGY+V *H. pylori* +SG+EV HSV-1 DSGY *P. gingivalis* DSG+EV *C. pneumoniae* DSGY V
8	S	*0.38*	*P. gingivalis* SGYEV *H. pylori* SGYE *C. neoformans* SGY++ *C. pneumoniae* SG+EV
9	G	*0.63*	*H. pylori* GYEVH Borrelia burgdorferi GYE V KL+ *C. neoformans* GYE LV and GY++ + LV *P. gingivalis* GYEV *C. pneumoniae* GYEV and GY HH
10	Y	*0.56*	*H. pylori* YE HH and YE+ HQ and Y++H Q and YE HHQ Cytomegalovirus YEVH Borrelia Burgdorferi YE+ KL *C. neoformans* YE + QK FC *P. gingivalis* Y++H H+K *C. pneumoniae* Y+V +Q LV
11	E	*0.58*	*H. pylori* EV +QK Cytomegalovirus EV HQ L Borrelia Burgdorferi EV +KL *C. neoformans* EV Q LV *P. gingivalis* EV KLV *C. pneumoniae* EV QKLV
12	V	0.35	*H. pylori*. +H QK Cytomegalovirus V HQ LV HHV-6 VH QK+V Borrelia Burgdorferi VH KL *C. neoformans* +HH LV *P. gingivalis* VH + LV *C. pneumoniae* V HQKL
13	H	−0.17	*H. pylori* and *C. pneumoniae* HHQK Cytomegalovirus HH KL *P. gingivalis* HH KL
14	H	−0.66	Borreli Burgdorferi HQKL+ *C. pneumoniae* and HSV-1 HQKL *P. gingivalis* +QKLV
15	Q	−1.03	*C. neoformans* and *P. gingivalis* and *C. pneumoniae* QKLV
16	K	−1.47	*H. pylori*: *Cryptococcus neoformans *Borrelia burgdorferi Chlamydophila pneumoniae KLVFF Human herpesvirus 1 KLVF
17	L	−1.34	Human herpesvirus 5: Human herpesvirus 6 LVFF
18	V	−1.20	
19	F	−0.93	
20	F	−0.98	
21	A	−0.82	
22	E	−0.31	
23	D	0.23	
**24**	**V**	*0.81*	Borrelia burgdorferi *Cryptococcus neoformans *Porphyromonas gingivalis VGSNK Cytomegalovirus +GSNK Helicobacter pylori Chlamydophila pneumoniae VGSN
**25**	**G**	*1.24*	*H. pylori* Chlamydophila pneumoniae GSNK
**26**	**S**	*1.22*	
**27**	**N**	*0.90*	
**28**	**K**	*0.36*	
29	G	0.30	
30	A	−0.24	
31	I	−0.58	
32	I	−1.00	
33	G	−1.14	
34	L	−1.19	
35	M	−1.23	
36	V	−1.16	69 viruses/phages VGGVV
37	G	−0.97	
38	G	−1.02	
39	V	−0.63	
40	V	−0.45	
41	I	−0.80	
42	A	−1.06	

**Table 1b:** (b)

NGF position	Amino acid	B-cell index	Alignment
18	A	*0.59*	*C. neoformans* AEPHS
19	E	*1.16*	*P. gingivalis* EPHSES—NVP Cytomegalovirus EPHS+S
20	P	*1.32*	*C. neoformans* P+S NVPAG and PHSES and P SESNV
21	H	*1.78*	Borrelia burgdorferi HSESN *C. neoformans* HSES VP and HSESN P H +P+
22	S	*1.64*	*C. neoformans* S+S VPAG T P Borrelia burgdorferi burgdorferi *C. neoformans* Chlamydophila pneumoniae *P. gingivalis* SESNV *P. gingivalis* S+SNVP *C. pneumoniae* SESNV A
23	E	*1.68*	*C. neoformans* ESNVP and ESNV AG
24	S	*1.51*	*C. neoformans* SNVPA
25	N	*1.61*	*C. neoformans* Cytomegalovirus *P. gingivalis* NVPAG *C. neoformans* NV TIPQA *P. gingivalis* +VPAG HT
26	V	*1.45*	*C. neoformans P. gingivalis* VPAGH *C. neoformans* VP AGHT *C. neoformans* VPAG TI and V AGHT+
27	P	*1.36*	*C. neoformans* HSV-1 PAGHT *C. neoformans* PAGHT P *C. neoformans* PAG TIP
28	A	*1.04*	*C. neoformans H. pylori* AGHTI *C. neoformans* AG H IPQA and AGHT PQ and AGHT+P and AG TIP HSV-1 HSV-2 AGH PQ and AGHT QA
29	G	*1.00*	*C. neoformans* GHTI Q and GHT PQ and G TIPQ
30	H	*0.93*	*C. pneumoniae* HTI QA *C. neoformans* HT PQA and HTIP A
31	T	*1.05*	*C. neoformans P. gingivalis* TIPQA
32	I	*0.95*	
33	P	*0.64*	
34	Q	*0.41*	
35	A	*0.46*	
51	A	*0.38*	*C. neoformans* AR SAPA and AR APAA and A SAPAA and ARSA AA and ARSAP A and ARS PAA and AR—SAPAA
52	R	*0.54*	HSV-1 RSAPAA Borrelia burgdorferi burgdorferri RSA AA
53	S	*0.91*	*C. neoformans C. pneumoniae* Cytomegalovirus HSV-1 HSV-2 *P. gingivalis* SAPAA
54	A	*0.89*	
55	P	*0.72*	
56	A	*0.69*	
57	A	*0.53*	
64	A	*0.46*	*C. neoformans* AG TRNI and AGQT RN and AGQTR *P. gingivalis* AGQTR
65	G	*0.59*	*C. neoformans* + RNITV and GQTRN
66	Q	*0.42*	*P. gingivalis* Borrelia burgdorferi QTRNI Cytomegalovirus QTRN—IT
67	T	*0.43*	*C. neoformans P. gingivalis* TRNIT
68	R	*0.49*	*C. neoformans* RNIT DP and RNITV
69	N	*0.76*	*C. neoformans C. pneumoniae H. pylori* NITVD
70	I	*0.66*	
71	T	*0.56*	
90	S	*0.40*	*C. neoformans* STQPPR and STQPP AA and STQPP EA *C. pneumoniae* STQ PRE *C. pneumoniae* Cytomegalovirus STQPP HSV-1 STQ PR
91	T	*0.63*	*C. neoformans* TQP REA and TQPPR
92	Q	*1.06*	*C. neoformans* QPPRE
93	P	*1.49*	*C. neoformans* PP REAA C Neoformans *P. gingivalis* PPREA
94	P	*1.91*	*C. neoformans* HSV-2 *H. pylori* PREAA
95	R	*2.13*	*C. neoformans P. gingivalis* REAAD and REAA TQ and RE ADT *C. neoformans* REAA DT and R AADT and R+AADT and READD HSV-1 REAA T Borrelia burgdorferi burgdorferi Cytomagalovirus RE ADT Cytomagalovirus REAA TQ *P. gingivalis* REA +TQ
96	E	*2.13*	*P. gingivalis* EAADTQ and EA TQDLD *C. neoformans* EAADT+ and EAAD QD and EAAD Q and EAA TQ
97	A	*1.92*	Borrelia burgdorferi burgdorferi *C. neoformans* AA TQD Borrelia burgdorferi burgdorferi *P. gingivalis* AAD QD HSV-2 AADT D HHV-6 AAD +DL *C. neoformans* AADT D and AA TQD and A DTQD and A TQDL and AADTQ and AADT+ D+D *P. gingivalis* ADTQ L *H. pylori* AADTQ
98	A	*1.9*	Borrelia burgdorferi ADT DLD *C. neoformans* AD QDLD and AD QDL *P. gingivalis* ADT DL and AD QDL *H. pylori* +TQDLD
99	D	*1.39*	*C. neoformans* DT DLD and D QDLD *C. pneumoniae* DTQDL
100	T	*1.25*	*C. neoformans H. pylori P. gingivalis* TQDLD
101	Q	*0.66*	Borrelia burgdorferi burgdorferi *C. neoformans H. pylori P. gingivalis* QDLDF *H. pylori* QD DFEV
102	D	*0.73*	Borrelia burgdorferi burgdorferi *C. neoformans H. pylori* DLDFE *C. neoformans* DLD EVG and DLDF VG
103	L	*0.54*	*C. neoformans* LD EVGG and LDFE GG and LDFEV and LDF VGG *H. pylori* LDF EVGG HSV-1 HSV-2 L+ EVGG
104	D	*0.45*	Borrelia burgdorferi burgdorferi DFEVG *C. neoformans* DF VGGA and D EVGGA
105	F	*0.42*	*C. neoformans* FEVGG Cytomegalovirus FE GGAA
106	E	*0.49*	*C. neoformans* EVGGAA and EVGGA P and EVGGA and EV GAAP and E+GGAAP *H. pylori* EVGGA Borrelia burgdorferi burgdorferi E+GG A PF+
107	V	*0.39*	*P. gingivalis* VGGAAP and VGGAA *C. neoformans* VGGAA PF and VGGAA NR and VGGAA and VG GAAP *C. pneumoniae* VGGA AP *H. pylori* VG GAAP and VGG APF and VGGAA *P. gingivalis* VGGAA
108	G	*0.74*	*C. neoformans C. pneumoniae* HSV-2 GGAAP
109	G	*0.44*	*C. neoformans* GA APFN T and GAAPF
110	A	*0.95*	*C. pneumoniae* AAPFN Borrelia burgdorferi AAP+NR HSV-1 AAP RT
111	A	*0.83*	*C. neoformans* APFN T RS *C. neoformans H. pylori* APFN TH *C. pneumoniae* AP N RT R RSSS
112	P	*0.99*	*C. neoformans* PFNRT and PF RTH Borrelia burgdorferi burgdorferi PF NRT
113	F	*0.84*	*C. neoformans* FNRT SKR
114	N	*0.75*	*C. neoformans* NR RSKRS S and NRT R RSSS Cytomegalovirus N T RSKRS
115	R	*0.63*	*C. neoformans* RT R SKRS and RTHRS Borrelia burgdorferi burgdorferi RTHRS
116	T	*0.63*	*C. neoformans* THRS RS and THRSK
117	H	*0.64*	*C. neoformans* HRS RSS and HRSKR C. Pneumonie HRSKR
118	R	*0.94*	*C. neoformans* RSKRSS and RS RSSS and RS KRSS and RS KRSS and RSK SSS and RSKRS S *P. gingivalis* RSKR S and RSKRS Borrelia burgdorferi burgdorferi RSKRS
119	S	*0.88*	*C. neoformans* SKRSSS and SKRSS *C. pneumoniae H. pylori* HSV-1 HSV-2 SKRSS
120	K	*1.12*	*C. neoformans* Cytomegalovirus HHV-6 *H. pylori* KRSSS
121	R	*1.13*	
122	S	*1.12*	
123	S	*0.83*	
124	S	*0.50*	
144	G	*0.52*	*C. neoformans* GDKTTA and GDKTT
145	D	*0.54*	*H. pylori* DK TATDI Borrelia burgdorferi burgdorferi *C. neoformans C. pneumoniae H. pylori* DKTTA HSV-1 +KTT TD
146	K	*0.84*	CF. Pneumoniae KTTAT+ *C. neoformans H. pylori* KTTAT
147	T	*1.27*	*C. neoformans* HSV-1 TTATDI *C. neoformans P. gingivalis* TTATD
148	T	*1.22*	*C. neoformans* TATDIK Borrelia burgdorferi burgdorferi *C. neoformans C. pneumoniae H. pylori* TATDI
149	A	*1.21*	*C. neoformans* HHV-6 HHV-6B *P. gingivalis* ATDIK
150	T	*1.08*	
151	D	*1.01*	
152	I	*0.97*	
153	K	*0.61*	
179	C	*0.87*	*C. neoformans* CR PNPV *C. pneumoniae* CRDPN P+ S RGI
180	R	*1.40*	*C. pneumoniae* RDPNPV Borrelia burgdorferi burgdorferi RD NP VDS *C. neoformans* RDP PVDS and RDPNP+ and RDPNP HHV-6 HHV-6B RDPNP HSV-1 HSV-2 RDPN V
181	D	*1.36*	Borrelia burgdorferi burgdorferi D NPVD and DPN VD *C. neoformans* DPNPV and DP PVD and DP PVDS and DPN VDS *C. pneumoniae* DPN VD HSV-1 DPNP S HSV-2 +P PVDS
182	P	*1.69*	*C. neoformans* PN VDSG and PNP DSG and PNPVD *P. gingivalis* PNPV+S and P PVDS
183	N	*1.72*	*H. pylori* NPVD G
184	P	*1.85*	*C. neoformans* PV DSGCR and P PVDS RG and PVDSG Cytomagalovirus P DSG RGI *P. gingivalis* PV DSGC
185	V	*1.74*	HSV-2 VDSG RG HSV-1 +DSG RG *C. neoformans* VDSG R
186	D	*1.51*	*C. neoformans* DSGC GI and DSG RG
187	S	*1.28*	Borrelia burgdorferi burgdorferi *C. neoformans* SGCRG and SG RGI and S CRGI
188	G	*0.79*	*C. neoformans* GC IDSKH W and GCRG D *P. gingivalis* GCRGI and GCRG+
189	C	*0.79*	*P. gingivalis* CRGID *C. neoformans* C GIDS
190	R	*0.85*	*C. neoformans* R IDSK and RGIDS and RGID K *H. pylori* RGIDS HSV-1 HSV-2 RG+DS *H. pylori* RG DSK Borrelia burgdorferi burgdorferi RGID K
191	G	*0.65*	*C. neoformans* GIDS HW and GIDSK and GIDS H Borrelia burgdorferi burgdorferi *H. pylori P. gingivalis* GIDSK *P. gingivalis* GID KH and G DSKH *H. pylori* GI SKH
192	I	*0.60*	Borrelia burgdorferi burgdorferi IDSKH *C. neoformans P. gingivalis* IDSK W
193	D	*0.3*	*C. neoformans* DSKH W Borrelia burgdorferi burgdorferi DSK WN
194	S	*0.40*	*C. neoformans* SKHW+
195	K	*0.44*	
197	W	*0.41*	
212	T	*0.33*	*C. neoformans* TMDGKQ and TMDGK
213	M	*0.31*	*P. gingivalis* MDGKQ
214	D	*0.39*	*C. neoformans* DGKQAA and DGKQA *C. pneumoniae* DGK AA
215	G	*0.48*	*C. neoformans* GKQAA
216	K	*0.53*	
217	Q	*0.45*	

**Table 1c:** (c)

Tau position	Amino acid	B-cell index	Alignment
53	E	*2.516*	*C. neoformans* (GSK3)EDGSEEP S and E+G EEPG and +DGS+EP S and ED GSEE GS and EDGS++ GS and EDGSE and +D EEPG and EDG S PGS Cytomagalovirus EDG EEP and +DG EE and ED GSEE *P. gingivalis* EDGSEE and E+G SEEP and EDGS EE HHV-6 HHV-6B EDGS EE *C. pneumoniae* E GSEE Borrelia burgdorferi E GSEE HHV-6 E GSEE
54	D	*2.568*	*C. neoformans* DGSEEP and DGS+EPG and DGSEE G *P. gingivalis* DGS EPGS and DGSEE and DGS EP and DGSE EP and DGSE P Cytomegalovirus DGS EP and DG EE G *C. pneumoniae* DGSE GS and +G EE GS Borrelia burgdorferi DGSE+ *P. gingivalis* +GSEE
55	G	*2.621*	*C. neoformans* GSEEP and G EEPG and G +EPGS and GS EEPG and GSEE G Cytomegalovirus GS+EP S *P. gingivalis* GS EPG *H. pylori* GSEE G *C. pneumoniae* GSEE GS
56	S	*2.732*	*C. neoformans* SEEPG and SEE GS and S EPGS and SEEP S *C. pneumoniae* SEEPG and SEE GS and SE PGS *P. gingivalis* SEE GS and S EPGS
57	E	*2.774*	*C. neoformans* EEPGS and +EPGS and EEPG E and EE GSE *C. pneumoniae* EEPGS HSV-1 HSV-2 EEPG
58	E	*2.678*	HHV-6 HHV-6B EPGSE *C. neoformans* EPGSE and EPGS+ *P. gingivalis* EPGS+
59	P	*2.564*	
60	G	*2.544*	
61	S	*2.556*	
62	E	*2.287*	
171	S	*2.315*	Borrelia burgdorferi SG GPED *C. neoformans* SG GPEDT and SGT PE and SGTGP and SG GPE and SGTG E and SG GP+ *P. gingivalis* SGTG E *C. pneumoniae* SGTG PE Cytomegalovirus SGTGP+
172	G	*2.54*	*C. neoformans* GT PED and GTG ED and GTGPE and G GPED and GTGP D and G EDTE HSV-1 GTGPE and GTGP D and GTGP+D HSV-2 GTGP D and G GPED *C. pneumoniae* GTGPE *H. pylori* GTGP D
173	T	*2.731*	HHV-6 TGPEDT and TG PEDT *C. neoformans* TG PEDT and TG EDT and T PEDT and TGPED *C. pneumoniae* TGPED *H. pylori* TG EDT Borrelia burgdorferi TGPE T+
174	G	*2.709*	*C. neoformans* GPEDT and GPED TE and GP DTE and GPED E HHV-6 HHV-6B TG PEDT
175	P	*2.807*	*C. neoformans H. pylori P. gingivalis* PEDTE
176	E	*2.7*	
177	D	*2.563*	
178	T	*2.397*	
179	E	*2.225*	
228	S	*2.396*	HSV-1 SP DSPP and SPQ SP *C. neoformans* SPQDS and S QDSP and SP DSPP and SP DSPP and SPQD PP and PQ DSPPS and SPQ PPSK and SPQ SP and SP DSP and SPQ SP Cytomegalovirus SP DSPP *P. gingivalis* SP DSP Borrelia burgdorferi SP++SPP and SP D PSK
229	P	*2.744*	*C. neoformans* PQ SPPS and P DSPPS and PQDSP and P+DSPP and PQ S PPSK and PQ SPP and PQD PP and P DSPP *P. gingivalis* PQDS P HSV-2 P DSPP HHV-6 PQ+ PP and PQ PP K Borrelia burgdorferi PQ+ P SK
230	Q	*2.763*	*C. neoformans* QDSPP and ++ PPSK and QDS PS and Q SPPS *P. gingivalis* QD PPS *C. pneumoniae* QDS PS
231	D	*2.929*	*C. neoformans* DSP PSK and DSPPS and DSP SK and D PPSK and +SPPS *P. gingivalis* DSP SK
232	S	*2.882*	*C. neoformans* SPPSK
233	P	*2.75*	
234	P	*2.794*	
235	S	*2.68*	
242	D	*2.387*	*C. neoformans* DGRPP *C. pneumoniae* DG PPQ HSV-1 DG PPQ HSV-2 DGRPP and DG PP+ Cytomegalovirus DG R PQ and DG PP and +GRPP Borrelia burgdorferi DG PP
243	G	*2.49*	*C. neoformans* GRPPQ
244	R	*2.56*	
245	P	*2.42*	
246	P	*2.164*	
247	Q	*2.004*	
331	P	*2.259*	*C. neoformans* PGEG PE and P EGPEA and PGEGP and PGEG EA and P EGPEA and PGEG E Cytomegalovirus PGEGP EA and PG GPE *C. pneumoniae* PGE PEA and PGEGP *H. pylori* PG GPE HSV-1 HHV-6 PG GP A
332	G	*2.681*	*C. neoformans* GEGPE and GEG EA and GE PEA and G GPEA and GEGP A and GEG EA and G+GPEA *C. pneumoniae* GE PEA *P. gingivalis* GEGPE and GEG EA
333	E	*2.715*	*C. neoformans P. gingivalis* EGPEA HSV-2 +GPEA
334	G	*2.634*	
335	P	*2.592*	
336	E	*2.463*	
337	A	*2.405*	
414	H	*1.819*	HSV-1 HSV-2 HPTPG *C. neoformans* HPT GSS and HP PGSS and HPTP SS and HP P SS *C. pneumoniae* HP PGS and +PT SS
415	P	*2.274*	Cytomegalovirus P PGSS and PTPG SS *C. pneumoniae* PT GSS and PTPG SS
416	T	*2.503*	
417	P	*2.717*	
418	G	*2.469*	
419	S	*2.054*	
420	S	*1.76*	
493	P	*2.43*	HSV-1 P APKTP and P APKTP and PPAP PP and PPA PP HSV-2 PPAP TPP Cytomegalovirus P APKT and PPAP PP and PP P PPS and PP KTPP and PP A TPPS and PPA TPP *C. neoformans* PPAP TPPS and PPAP KTP S and PP PKTP and PP PKTP and PPAPKT and PPAP KT *P. gingivalis* PPAPK P *C. pneumoniae* PPAPK and PP PKT and PP AP PP and PP TPP Borrelia burgdorferi PAP T PS and PPA KTP and PP P TPP
494	P	*2.77*	HSV-1 PAP KTP and PA KTPP HSV-2 PAPK PP HHV-6 PAP PPS *C. neoformans* PAPKTP S and PAPK PP *P. gingivalis* PAPKT *C. pneumoniae* PAP TP and PAPK P
495	A	*2.94*	*C. neoformans* AP PPS *P. gingivalis* A KTPPS HHV-6 APKTP
496	P	*2.89*	*C. neoformans* PKTPPS and PKTP PS and PK TPPS *P. gingivalis* PKT PS
497	K	*2.95*	*C. neoformans* KTPPS
498	T	*2.98*	
499	P	*2.69*	
500	P	*2.51*	
501	S	*2.343*	
502	S	*2.123*	
